# Extracellular signal-Regulated Kinase 5 (ERK5) is required for the Yes-associated protein (YAP) co-transcriptional activity

**DOI:** 10.1038/s41419-023-05569-7

**Published:** 2023-01-17

**Authors:** Francesca Ippolito, Veronica Consalvi, Valeria Noce, Cecilia Battistelli, Carla Cicchini, Marco Tripodi, Laura Amicone, Alessandra Marchetti

**Affiliations:** 1 Department of Molecular Medicine, Sapienza University of Rome, Rome, Italy; 2 National Institute for Infectious Diseases L. Spallanzani, IRCCS, Rome, Italy

**Keywords:** Cell signalling, Cancer

## Abstract

YES-associated protein (YAP) is a transcriptional cofactor with a key role in the regulation of several physio-pathological cellular processes, by integrating multiple cell autonomous and microenvironmental cues. YAP is the main downstream effector of the Hippo pathway, a tumor-suppressive signaling able to transduce several extracellular signals. The Hippo pathway acts restraining YAP activity, since its activation induces YAP phosphorylation and cytoplasmic sequestration. However, recent observations indicate that YAP activity can be also modulated by Hippo independent/integrating pathways, still largely unexplored. In this study, we demonstrated the role of the extracellular signal-regulated kinase 5 (ERK5)/mitogen-activated protein kinase in the regulation of YAP activity. By means of ERK5 inhibition/silencing and overexpression experiments, and by using as model liver stem cells, hepatocytes, and hepatocellular carcinoma (HCC) cell lines, we provided evidence that ERK5 is required for YAP-dependent gene expression. Mechanistically, ERK5 controls the recruitment of YAP on promoters of target genes and its physical interaction with the transcriptional partner TEAD; moreover, it mediates the YAP activation occurring in cell adhesion, migration, and TGFβ-induced EMT of liver cells. Furthermore, we demonstrated that ERK5 signaling modulates YAP activity in a LATS1/2-independent manner. Therefore, our observations identify ERK5 as a novel upstream Hippo-independent regulator of YAP activity, thus unveiling a new target for therapeutic approaches aimed at interfering with its function.

## Introduction

YAP (Yes-associated protein, Yki ortholog) is a transcriptional cofactor involved in the regulation of various processes, including cell proliferation, differentiation, tissue regeneration, and mass homeostasis of organs and tissues, by integrating multiple cell autonomous and microenvironmental cues [1]. Moreover, hyperactivation of YAP has been observed in several human cancers where it is associated with the acquisition of stemness and metastatic properties, chemoresistance, increased cell proliferation and survival [2].

Hippo pathway has been described as the main regulator of YAP activity in response to a variety of intrinsic and microenvironmental signals [3]. The regulatory module of Hippo includes the kinases MST1/2 and LATS1/2. When Hippo is activated, the LATS-mediated phosphorylation of YAP prevents both its stability and nuclear translocation; on the contrary, when Hippo signaling is inhibited by specific extracellular signals or genetic alterations, YAP translocates into the nucleus where it drives several transcriptional programs in a context-dependent manner [4]. In recent years, the wide complexity of the YAP activity regulation has been unveiled and several signaling pathways, independent of or interacting with the Hippo pathway, were found involved. In particular, elements belonging to other cancer-relevant pathways, especially G protein-coupled receptors (GPCRs), transforming growth factor-β (TGFβ) and Wnt pathways, have been shown to play an important role in YAP subcellular distribution and activity [4]. Notably, it has been recently described a significant Hippo-independent role of some cytoplasmic and nuclear serine/threonine kinases, mainly accomplished by direct phosphorylation of YAP or of its functional cofactors [5–9]. A role of some MAPKs (i.e. p38, JNK, and ERK1/2) in the regulation of YAP activity has been also reported [10–12]. The dissection of additional levels of regulation of YAP activity, particularly in cancer and stem cells, is required for the identification of novel therapeutic targets for the treatment of YAP-dependent cancers as well as new possible tools in regenerative medicine.

In this study, we specifically investigated the role of extracellular signal-regulated kinase 5 (ERK5) in the regulation of YAP activity in liver cells.

ERK5 is a serine/threonine kinase belonging to the family of conventional mitogen-activated protein kinases (MAPKs), involved in the transduction of a large variety of stimuli (including mitogens, hormones, cytokines, neurotransmitters, oxidative and osmotic stresses as well as mechanical stimuli) and able to induce different cell responses (mainly proliferation, differentiation, migration, and protection from apoptosis) [13]. The ERK5 upstream signaling includes MEKK2/MEKK3 and MEK5 kinases. Notably, while MEKK2/MEKK3 can also modulate other MAPK pathways, MEK5 specifically activates ERK5; for this reason, any cellular function of MEK5 is usually linked to its ability to phosphorylate ERK5 [14]. The phosphorylation by MEK5 is responsible for ERK5 kinase activation and for its nuclear translocation. Into the nucleus, ERK5 regulates gene expression not only indirectly through the phosphorylation of specific transcription factors, but also by acting as transcriptional cofactor [15]. ERK5 protein, indeed, harbors a unique and large non-kinase C-terminus, containing a transactivation domain, required to increase the transcriptional activity of target proteins [16].

Although ERK5 shares a series of cytoplasmic and nuclear substrates with other MAPKs [17], its role is not redundant, neither during development (ERK5-knockout animals die during development) [18–20] nor in the adult, where ERK5 deficiency significantly affects muscle differentiation, proliferation/function of endothelial cells and vasculogenesis, function of the immune system [21]. Interestingly, in the last few years, accumulating lines of evidence highlighted the key role of ERK5 in the onset and progression of several types of cancer, where it contributes to sustain cell proliferation and survival, to evade the immune system, to promote angiogenesis and tumor-associated inflammation, to support cell invasion and metastasis [22]. In particular, a direct involvement of ERK5 in hepatocellular carcinoma (HCC) has been established, where aberrant activation of MEK5/ERK5 signaling and *ERK5/MAPK7* gene amplification have been reported and correlated with tumor progression and poor prognosis [23–25].

Recently, a body of correlative evidence suggests a functional link between ERK5 and YAP. Firstly, ERK5 can mediate the signal transduction from mechanical stresses (i.e. fluid shear stress and stretching) [26–29], where the YAP involvement is well known [30]. Secondly, ERK5 has a role in the epithelial-to-mesenchymal transition (EMT) [31], a process of cellular plasticity, aberrantly activated in cancer, that promotes tumor progression toward a malignant phenotype [32]. Notably, YAP is a further crucial player of the EMT, during which it is significantly upregulated. In particular, recent reports showed that YAP acts as a primary mediator of the EMT triggered by TFGβ [33, 34] and is required for the SMAD2/3 nuclear translocation [35]. Moreover, we demonstrated that both ERK5 and YAP are positive regulators of the EMT master transcription factor Snail. Interestingly, recent evidence demonstrated a key role of both YAP and ERK5 also in the regulation of the embryonic stem cell (ESC) identity: overexpression of YAP in human embryonic stem cells promotes the generation of naive pluripotent stem cells [36]; ERK5 is required for the maintenance of ESC in the naïve state and for the inhibition of specific differentiation programs [37]. Furthermore, the MEK5/ERK5 pathway activation by YAP in promoting muscle cell differentiation has been recently reported [38]. Finally, as said above, both ERK5 and YAP were shown to play crucial roles in tumor growth and progression of several kinds of human cancers, including hepatocellular carcinoma [2, 22].

Here, we demonstrated for the first time that ERK5 activity is required for YAP-dependent gene expression in liver progenitor cells and in human hepatocellular carcinoma cell lines and, mechanistically, for the interaction of YAP with its main transcriptional partner TEAD and, consequently, for its recruitment on target gene promoters. Furthermore, we gathered evidence that MEK5/ERK5 signaling can modulate YAP activity in a LATS1/2-independent manner. Therefore, our results identify ERK5 as a novel Hippo-independent regulator of YAP activity, thus unveiling a new target for mono- or combination therapies aimed at interfering with YAP function.

## Results

### ERK5 is required for gene expression driven by endogenous or overexpressed YAP in liver cells

**Fig. 1:**
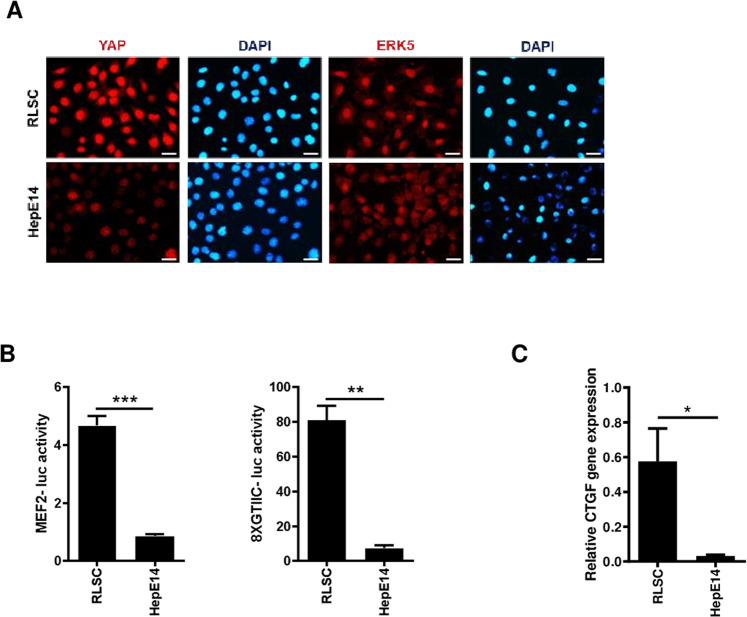
ERK5 and YAP activity are directly correlated in liver cell differentiation. **A** Immunofluorescence analysis of RLSC and HepE14 cell lines. Cells were stained with anti-YAP or anti-ERK5 antibodies (red) and DAPI (nuclei, blue). Scale bar: 50 µm. **B** Luciferase assay. MEF2-luc or 8xGTIIC-luc reporters were transiently co-transfected in RLSC and HepE14 cell lines, together with a Renilla expression vector. Luciferase activities were normalized for Renilla luciferase activity and expressed as arbitrary units. Statistically significant differences are reported (**p < 0.01; ***p < 0.001). **C** RT-qPCR analysis of *Ctgf* gene expression in RLSC and HepE14 cell lines. The values are calculated by the 2(−ΔCt) method and shown as means ± S.E.M. of at least three independent experiments. Statistically significant differences are reported (*p < 0.05).

To assess the hypothesized functional link between ERK5 and YAP proteins, we firstly evaluated ERK5 activity in liver cell models where the transcriptional activation of YAP was previously characterized [39]. Therefore, we utilized RLSC precursor/stem cells, expressing high levels of nuclear and functionally active YAP protein, and HepE14 differentiated hepatocytes, showing only a residual presence of YAP protein, predominantly cytoplasmic and transcriptionally inactive (Fig. 1A, left panels, and Fig. 1B). As shown in Fig. 1A (right panels), the localization of ERK5, predominantly nuclear in RLSCs and diffused in HepE14 cells, was found to correlate with that of YAP. To evaluate the activity of the two proteins, two constructs have been used in luciferase assays, an ERK5-responsive construct where the reporter gene is under the control of a promoter responsive to the transcription factor MEF2, the main positive target of ERK5 kinase activity (MEF2-luc reporter) [40] and a YAP-responsive construct (Supplementary Fig. S1A) where the reporter gene is under the control of a synthetic promoter containing eight binding sites for TEAD, the main transcriptional cofactor of YAP (8XGTIIC-luc reporter) [41]. As expected, the luciferase activity of the YAP-responsive construct, as well as the expression of YAP target gene *Ctgf*, was found at a higher level in RLSCs than in HepE14 (Fig. 1B, right panel, and Fig. 1C). Interestingly, the luciferase activity of the ERK5-responsive construct in the same cell lines resulted strictly correlated with the YAP-dependent transcriptional activity (Fig. 1B, left panel).

**Fig. 2:**
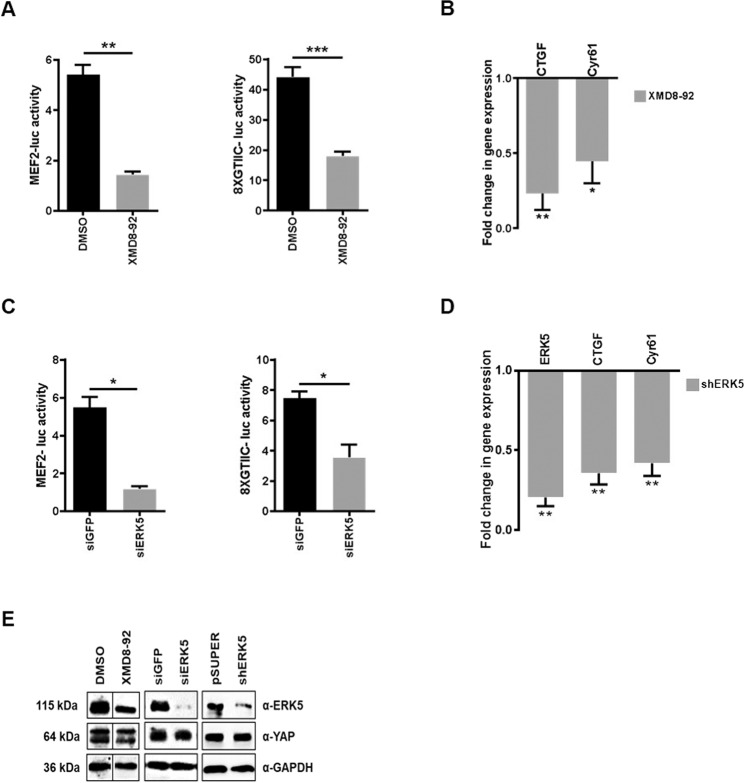
ERK5 is required for the steady-state YAP transcriptional activity. **A** Luciferase assay. MEF2-luc or 8xGTIIC-luc reporters were transiently co-transfected in RLSCs, together with a Renilla expression vector. Luciferase activities were normalized for Renilla luciferase activity and expressed as arbitrary units. Twenty-four hours post-transfection, cells were treated with 10 µM XMD8-92 or its solvent DMSO for 16 h. Statistically significant differences are reported (**p < 0.01; ***p < 0.001). **B** RT-qPCR analysis of the indicated genes in RLSCs treated with XMD8-92 or DMSO. The values are calculated by the 2(−ΔCt) method, expressed as fold change in gene expression versus the control (DMSO, arbitrary value = 1) and shown as means ± S.E.M. of at least three independent experiments. Statistically significant differences are reported (*p < 0.05; **p < 0.01). **C** Luciferase assay. MEF2-luc or 8xGTIIC-luc reporters were transiently co-transfected in RLSCs, together with a Renilla expression vector and siERK5 or siGFP. Luciferase activities were normalized for Renilla luciferase activity and expressed as arbitrary units. Statistically significant differences are reported (*p < 0.05). **D** RT-qPCR analysis of the indicated genes in RLSCs stably transfected with pSUPER or pSUPER-shERK5 vector. The values are calculated by the 2(−ΔCt) method, expressed as fold change in gene expression versus the control (pSUPER, arbitrary value = 1) and shown as means ± S.E.M. of at least three independent experiments. Statistically significant differences are reported (**p < 0.01). **E** Western blot for the indicated proteins in RLSCs as in (**A**), (**C**), and (**D**). GAPDH has been utilized as loading control. WB images represent one indicative experiment of at least three independent ones.

**Fig. 3:**
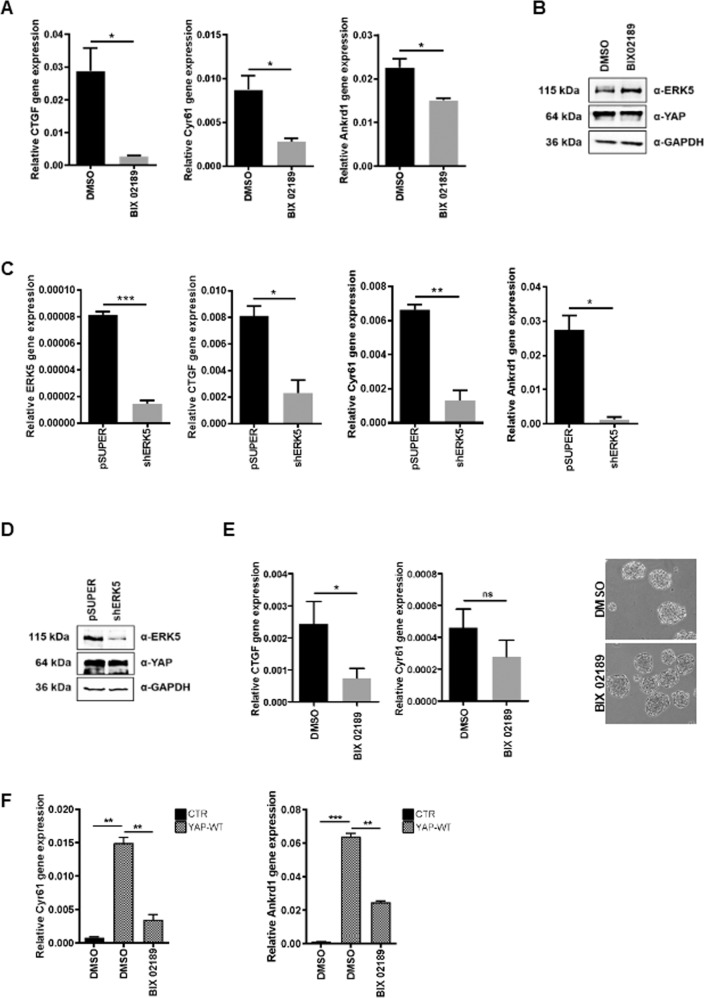
ERK5 is required for endogenous and exogenous YAP transcriptional activity in HuH7. **A** RT-qPCR analysis of the indicated genes in HuH7 treated with 10 µM BIX02189 or DMSO. Data are expressed as relative gene expression and shown as mean ± S.E.M. of three independent experiments. Statistically significant differences are reported (*p < 0.05). **B** Western blot for the indicated proteins in HuH7 as in (**A**). GAPDH has been utilized as loading control. **C** RT-qPCR analysis of the indicated genes in HuH7 transfected with pSUPER or pSUPER-shERK5 vector. Data are expressed as in (**A**). Statistically significant differences are reported (*p < 0.05; **p < 0.01; ***p < 0.001). **D** Western blot for the indicated proteins in HuH7 as in (**C**). GAPDH has been utilized as loading control. **E** RT-qPCR analysis of the indicated genes in HuH7 cells, grown as spheroids for 24 h and treated with 10 µM BIX02189 or DMSO. Data are expressed as relative gene expression and shown as mean ± S.E.M. of three independent 3D cell cultures. Statistically significant differences are reported (unpaired, one-tailed Student’s *t* test; *p < 0.05; ns= not significant). Representative images of spheroids in the two different cell conditions are shown. **F** RT-qPCR analysis of the indicated YAP target genes in wild-type YAP-overexpressing HuH7 cells (YAP-WT) and in control cells (CTR), treated with 10 µM BIX02189 or DMSO. Data are expressed as relative gene expression and shown as mean ± S.E.M. of three independent experiments. Statistically significant differences are reported (**p < 0.01; ***p < 0.001).

To evaluate whether the ERK5 function was only correlative or rather causal to the regulation of YAP activity, experiments of ERK5 inactivation/silencing were performed in RLSCs. For ERK5 kinase inactivation, we took advantage of two commercial and well-characterized chemical inhibitors, the MEK5 inhibitor BIX02189 [42] and the ERK5 inhibitor XMD8-92 [43]. In RLSCs, where both ERK5 and YAP activity has been observed (Fig. 1), the treatment with XMD8-92 induced YAP functional impairment. In fact, together with a downregulation of the MEF2-Luciferase activity, demonstrating the effective inhibition of ERK5, a significant decrease of both the YAP-responsive reporter activity (Fig. 2A) and the transcription of YAP target genes *Ctgf* and *Cyr61* (Fig. 2B) were observed. Similar results were obtained both with the other MEK5/ERK5 inhibitor BIX02189 (Supplementary Fig. S1B) and after the ERK5 knockdown, achieved by means of siRNAs or shRNAs expression (Fig. 2C, D, respectively). Notably, YAP protein level was not affected by chemical ERK5 inactivation nor by ERK5 knockdown (Fig. 2E). Furthermore, the dependence of YAP activity on ERK5 has been also demonstrated in human hepatoma cells. As shown in Fig. 3, the chemical inhibition of MEK5/ERK5 obtained with BIX02189 (Fig. 3A, B) and the genetic silencing of the kinase by shERK5 (Fig. 3C, D) significantly downregulated YAP-dependent transcription in HuH7 cells. Similar results have been obtained in a second human hepatoma cell line, HepG2 (Supplementary Fig. S2A, B). Moreover, the BIX02189 efficacy has been tested also in HuH7 cells grown as suspended aggregate/spheroids. In this experimental condition a downregulation of the YAP target genes *CTGF* and *CYR61* can be observed, although, as expected, at less significant level compared to that obtained in 2D cell culture (Fig. 3E). Furthermore, the effect of ERK5 inhibition on the transcriptional activity of YAP was confirmed in HuH7 cells ectopically expressing a wild-type form of YAP protein (Fig. 3F and Supplementary Fig. S3A, B, left panels). The YAP-induced transcriptional activation (i.e. upregulation of *CYR61* and *ANKRD1* target genes), indeed, was strongly impaired by treatment of the cells with the MEK5/ERK5 chemical inhibitor BIX02189.

Of note, at the doses utilized (chosen on the basis of the cell-specific IC50 and the most significant inhibition of ERK5 activity assessed by the MEF2-luc reporter activity in RLSC and HuH7 cell lines), MEK5/ERK5 inhibitors did not significantly impact cell proliferation/vitality (Supplementary Fig. S4).

Overall, these data demonstrate that ERK5 activity is required for YAP-driven gene expression in liver stem/progenitor cells and in hepatoma cells.

### ERK5 activation is sufficient to promote YAP transcriptional activity

To further demonstrate the role of ERK5 in the regulation of YAP activity, we performed experiments of ERK5 overexpression/activation.

**Fig. 4:**
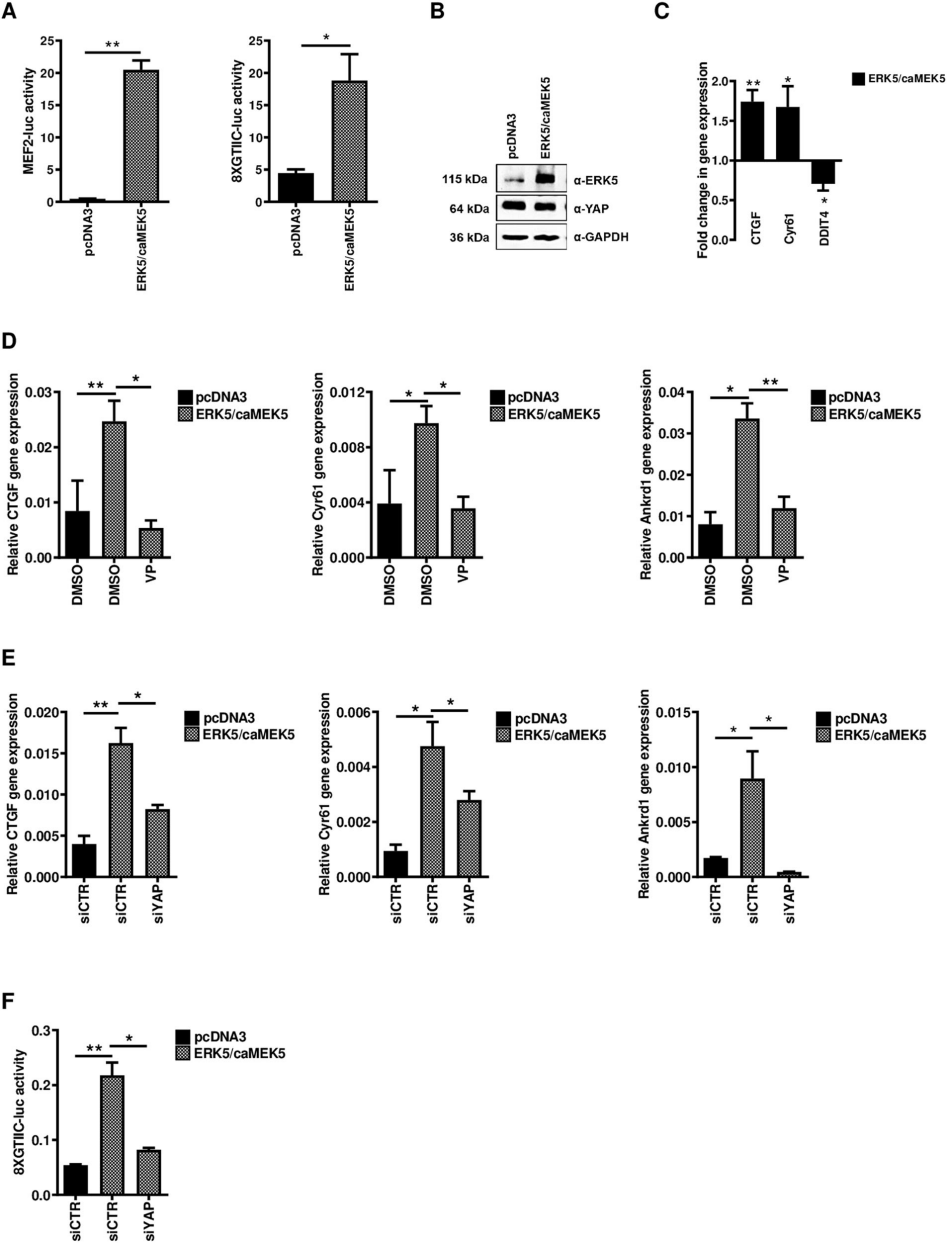
ERK5 constitutive activation promotes YAP transcriptional activity. **A** Luciferase assay. MEF2-luc or 8xGTIIC-luc reporters were transiently co-transfected in HepE14 cells, together with a Renilla expression vector, an ERK5 and a constitutive active MEK5 expressing vector (ERK5/caMEK5) or the empty vector (pcDNA3). Luciferase activities were normalized for Renilla luciferase activity and expressed as arbitrary units. Statistically significant differences are reported (*p < 0.05; **p < 0.01). **B** Western blot for the indicated proteins in ERK5/caMEK5-overexpressing HepE14. GAPDH has been utilized as loading control. WB images represent one indicative experiment of three independent ones. **C** RT-qPCR analysis of the indicated YAP target genes in ERK5/caMEK5-overexpressing RLSCs. The values are calculated by the 2(−ΔCt) method, expressed as fold change in gene expression versus the control (empty vector, arbitrary value = 1) and shown as means ± S.E.M. of at least three independent experiments. Statistically significant differences are reported (*p < 0.05; **p < 0.01). **D** RT-qPCR analysis of the indicated YAP target genes in ERK5/caMEK5-overexpressing and in control HuH7 cells, treated with 10 µM of YAP-TEAD inhibitor Verteporfin (VP) or with DMSO. The values are calculated by the 2(−ΔCt) method and shown as means ± S.E.M. of three independent experiments. Statistically significant differences are reported (*p < 0.05; **p < 0.01). **E** RT-qPCR analysis of the indicated YAP target genes in YAP-silenced ERK5/caMEK5-overexpressing HuH7 cells (siYAP), compared with cells transfected with control siRNAs (siCTR). The values are calculated by the 2(−ΔCt) method and shown as means ± S.E.M. of three independent experiments. Statistically significant differences are reported (*p < 0.05; **p < 0.01). **F** Luciferase assay. 8xGTIIC-luc reporter was transiently co-transfected in HuH7 cells, together with a Renilla expression vector, an ERK5 and a constitutive active MEK5 expressing vector (ERK5/caMEK5) or the empty vector (pcDNA3), in the presence of siYAP or siCTR. Luciferase activities were normalized for Renilla luciferase activity and expressed as arbitrary units. Statistically significant differences are reported (*p < 0.05; **p < 0.01).

The constitutive activity of ERK5 has been obtained by transient transfection in HepE14 hepatocytes of an ERK5 expressing vector together with a construct expressing a constitutively active mutant of the upstream kinase MEK5 (caMEK5) [44]. As shown in Fig. 4A, ERK5/caMEK5-overexpression induced in HepE14 a significant increase of the YAP-responsive reporter activity, indicating a functional activation of YAP by ERK5. Of note, ERK5 overexpression, as well as ERK5 knockdown, did not affect YAP expression at the protein level (Fig. 4B). However, the modulation of YAP target genes cannot be observed (data not shown), suggesting that ERK5 activation is not sufficient to promote YAP-dependent gene expression in HepE14. This result could be related to the differentiated state of these cells where the chromatin state/configuration and the endogenous level of YAP protein could not permit the expression of YAP target genes by ERK5. This hypothesis has been supported by the results obtained in RLSC liver progenitors where the constitutive activation of MEK5/ERK5 signaling induced the upregulation of *Ctgf* and *Cyr61* and the downregulation of *Ddit4*, positive and negative YAP-target genes, respectively (Fig. 4C). Similar results have been obtained in HuH7 cells overexpressing caMEK5/ERK5 where a significant upregulation of the canonical YAP target genes *CTGF*, *CYR61*, and *ANKRD1* has been observed (Fig. 4D). Notably, both the treatment of the cells with the YAP inhibitor Verteporfin and the genetic silencing of *YAP* inhibited the ERK5-dependent upregulation of YAP target genes (Fig. 4D, E) and the YAP-dependent transcriptional activity (Fig. 4F), thus excluding a YAP-independent regulation of these genes by the kinase.

Overall, these data demonstrate that the constitutive ERK5 activation is sufficient to promote YAP transcriptional activity and YAP-dependent gene expression in permissive liver cells.

### ERK5 activity is required for YAP/TEAD interaction and for YAP recruitment on DNA

**Fig. 5:**
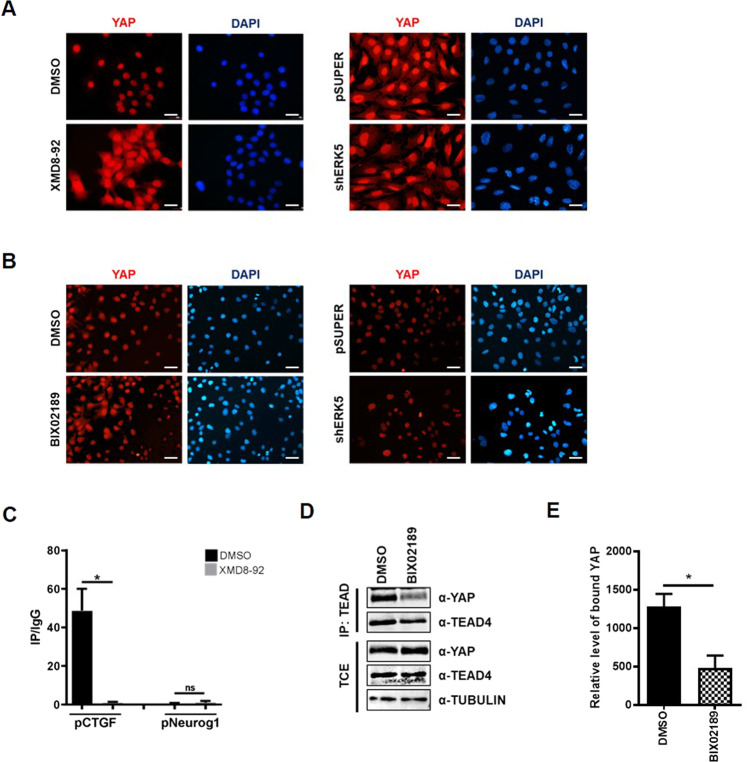
ERK5 activity is required for the recruitment of YAP on target gene promoters and for YAP/TEAD interaction. **A** Immunofluorescence analysis of RLSC treated with XMD8-92 or with DMSO for 16 h (left panels) and of RLSC transfected with shERK5 or with the empty vector (right panels). Cells were stained with an anti-YAP antibody (red) and DAPI (nuclei, blue). Images are representative of three independent experiments. Scale bar: 50 µm. **B** Immunofluorescence analysis of HuH7 treated with BIX02189 or with DMSO for 16 h (left panels) and of HuH7 transfected with shERK5 or with the empty vector (right panels). Cells were stained with an anti-YAP antibody (red) and DAPI (nuclei, blue). Images are representative of three independent experiments. Scale bar: 50 µm. **C** qPCR analysis of ChIP assays with anti-YAP antibody (IP) and, as control, normal rabbit IgG (IgG) on chromatin from RLSC treated with XMD8-92 or with DMSO for 16 h. The TEAD consensus region embedded in the *Ctgf* gene promoter was analyzed. A YAP unbounded region of *Neurogenin 1* promoter was utilized as negative control. Data are normalized to total chromatin input and background (control immunoprecipitation with IgG) and expressed as IP/IgG. Mean ± SEM of qPCR data obtained in triplicate from three independent experiments is reported. Statistical significance: *p < 0.05; ns = not significant. **D** Co-immunoprecipitation of YAP and TEAD proteins. Total cell extracts (TCEs) and anti-TEAD4 immunoprecipitates (IP) were analyzed by immunoblotting with anti-YAP and anti-TEAD4 antibodies. Tubulin has been utilized as loading control of TCEs. WB images represent one indicative experiment of three independent ones. **E** anti-YAP IP from three independent experiments was quantified by densitometric analysis and normalized on the relative anti-TEAD4 IP. Statistical significance: *p < 0.05.

The data reported so far showed that ERK5 is necessary for the transcriptional activity of YAP and its overexpression sufficient to trigger a YAP-dependent gene expression. To investigate the molecular mechanisms underlying the regulation of YAP by ERK5, we first analyzed the subcellular localization of YAP in the RLSC and in HuH7 cell lines following ERK5 chemical inhibition or genetic silencing. As shown in Fig. 5A, B, although the YAP immunostaining results improved both by transfection procedure and XMD8-92 treatment in RLSCs (without modification of protein amount, as shown in Fig. 2E), the nuclear localization of YAP appeared unaffected by ERK5 inhibition or silencing (left and right panels, respectively). Therefore, we next analyzed the DNA occupancy of YAP on specific chromatin sites in conditions of ERK5 activity inhibition. A ChIP assay was performed with an anti-YAP antibody on chromatin from RLSC cell line treated with the ERK5 inhibitor XMD8-92 or with its solvent DMSO. The TEAD binding site in the *CTGF* promoter, previously described as able to recruit YAP in these cells [39], has been explored by qPCRs. As negative control of YAP recruitment, the regulatory region of the *Neurogenin 1* gene has been assessed. As shown in Fig. 5C, the lack of YAP binding on DNA was observed following ERK5 inactivation, thus indicating that ERK5 activity is required for the recruitment of YAP on target gene promoters. Interestingly, co-immunoprecipitation assays in HuH7 cells treated with a chemical inhibitor of ERK5 revealed that ERK5 inactivation reduced the interaction between YAP and TEAD4 (Fig. 5D, E) without affecting TEAD4 protein expression (Fig. 5D). This result suggests that the failure of the YAP recruitment on chromatin can be mediated by the loss of the interaction between YAP and TEAD.

Altogether, these results indicate that ERK5 positively regulates YAP transcriptional activity by controlling its recruitment on target gene promoters and the interaction with TEAD.

### ECM-induced YAP activation requires ERK5 activity

In the attempt to identify the cellular processes involving an ERK5-dependent YAP activation, we first analyzed the effect of ERK5 inhibition in cell-extracellular matrix (ECM) adhesion dynamics where the modulation of transcriptional activity of YAP has been well documented, both in the physiological and pathological cell response to mechanical stimuli as well as in cancer progression [45]. In particular, it has been previously reported that YAP activity is inhibited upon cell detachment and upregulated by cell adhesion [46].

**Fig. 6:**
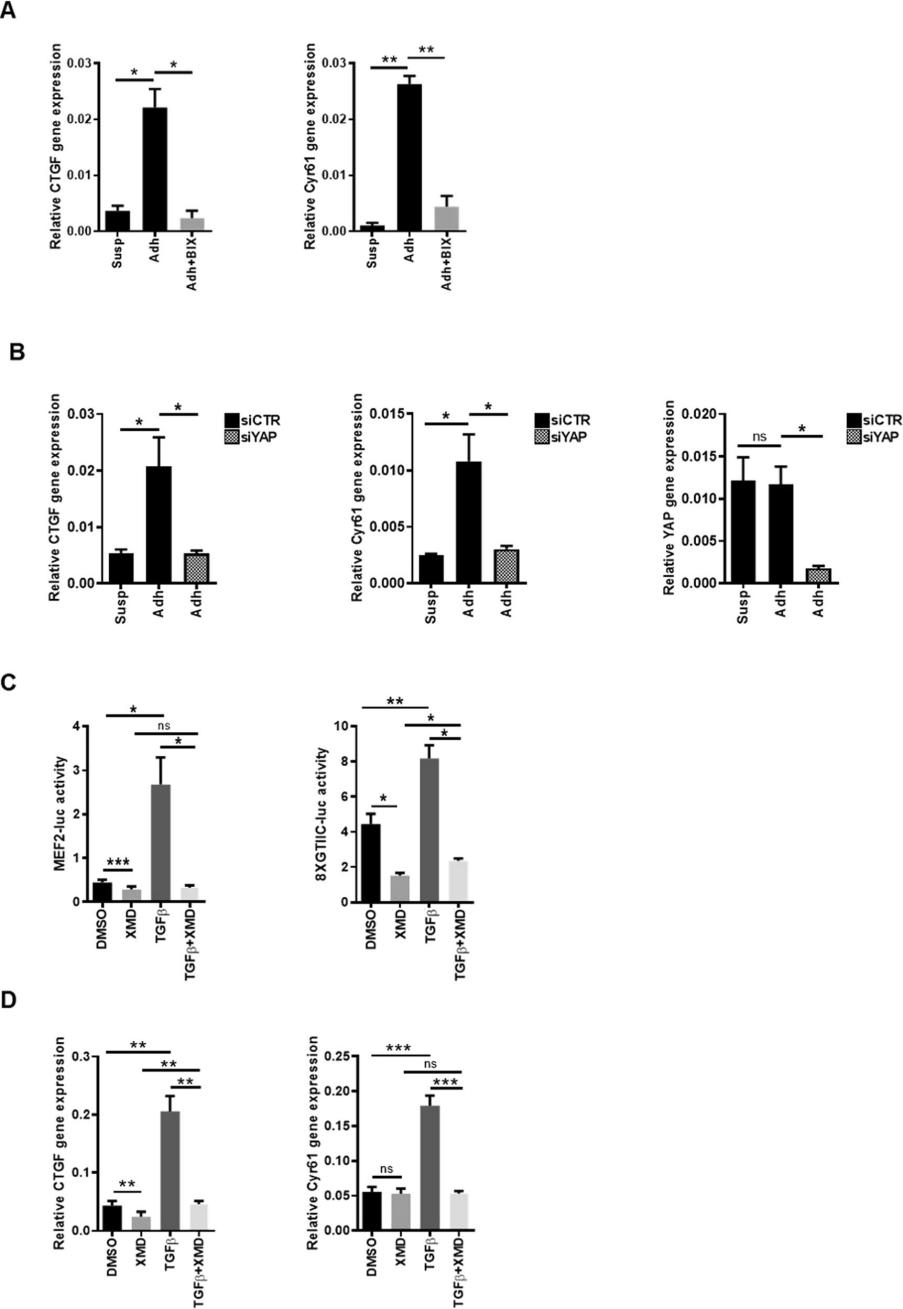
Adhesion- and TGFβ- mediated activation of YAP requires ERK5. **A** RT-qPCR analysis of the indicated genes in HuH7. Cells were trypsinized, maintained in suspension for 10’ and collected (Susp) or plated for 3 h on collagen-coated plates (Adh) in the presence or absence of ERK5 inhibitor BIX02189. Data are expressed as relative expression and shown as means ± S.E.M. of at least three independent experiments. Statistically significant differences are reported (*p < 0.05; **p < 0.01). **B** RT-qPCR analysis of the indicated genes in suspended and adherent YAP-silenced HuH7 (siYAP), compared with cells transfected with control siRNAs (siCTR). Data are expressed as relative expression and shown as means ± S.E.M. of at least three independent experiments. Statistically significant differences are reported (*p < 0.05; ns =not significant). **C** Luciferase assay. MEF2-luc or 8xGTIIC-luc reporters were transiently co-transfected in HepE14, together with a Renilla luciferase expression vector. Twenty-four hours post-transfection, cells were treated with TGFβ1 or left untreated for 24 h, in the presence of 10 µM XMD8-92 or DMSO. Luciferase activities were normalized for Renilla luciferase activity and expressed as arbitrary units. Statistically significant differences are reported (*p < 0.05; **p < 0.01; ***p < 0.001; ns = not significant). **D** RT-qPCR analysis of *Ctgf* and *Cyr61* gene expression in untreated or TGFβ1-treated HepE14 cells. The values are calculated by the 2(−ΔCt) method and shown as means ± S.E.M. of at least three independent experiments. Statistically significant differences are reported (**p < 0.01; ***p < 0.001; ns = not significant).

Starting from these observations, we investigated the effect of ERK5 inhibition on YAP target gene expression in cell detachment and adhesion dynamics. Therefore, trypsinized Huh7 cells were maintained in suspension for 10′ and collected or re-plated on collagen-coated dishes and maintained in adhesion for 3 h, in the presence of MEK5/ERK5 chemical inhibitor BIX02189 or DMSO. As expected, in suspended cells a low level of YAP transcriptional activity was observed while cell adhesion induced YAP-dependent gene expression (Fig. 6A, B). The treatment of cells with BIX02189 significantly impaired the upregulation of YAP target genes *CTGF* and *CYR61* induced by cell adhesion to ECM (Fig. 6A). Similar results were obtained in HepG2 cells (Supplementary Figure S2C).

Altogether, these data indicate that cell/ECM interactions lead to an ERK5-dependent YAP activation.

### ERK5 mediates the TGFβ-induced YAP activation

Several lines of research unveiled a multilevel crosstalk between Hippo/YAP signaling and TGFβ pathway. However, while it has been shown that TGFβ-induced SMAD nuclear translocation is dependent on YAP [35] and that YAP knockdown strongly impacts on the cell response to the cytokine in terms of apoptosis and EMT [33], how the cytokine can control the YAP activity has not yet been elucidated.

We previously demonstrated that ERK5 is activated in the TGFβ-induced EMT of HepE14 hepatocytes and plays a crucial role in the Snail cytoplasmic stabilization [47]. We therefore asked whether ERK5 could also mediate YAP activation in hepatocytes in response to TGFβ treatment. To this aim, we analyzed the YAP transcriptional activity in TGFβ-treated cells in the presence of ERK5 chemical inhibition.

As shown in Fig. 6, in HepE14 treated with the cytokine, the expected ERK5 activation (Fig. 6C, left panel) correlated with the YAP functional activation, assessed both by the activity of YAP-responsive luciferase reporter 8XGTIIC-luc (Fig. 6C, right panel) and by the expression of its target genes, *Ctgf* and *Cyr61* (Fig. 6D). The TGFβ-induced transcriptional activity of YAP is ERK5-dependent since the treatment of cells with XMD8-92 impaired both YAP-dependent luciferase activity and target gene expression.

Overall, these results demonstrate that ERK5 is required for the YAP transcriptional activation induced by TGFβ in hepatocytes undergoing EMT.

### YAP-induced motility of liver cancer cells requires the LATS1/2-independent ERK5 activity

**Fig. 7:**
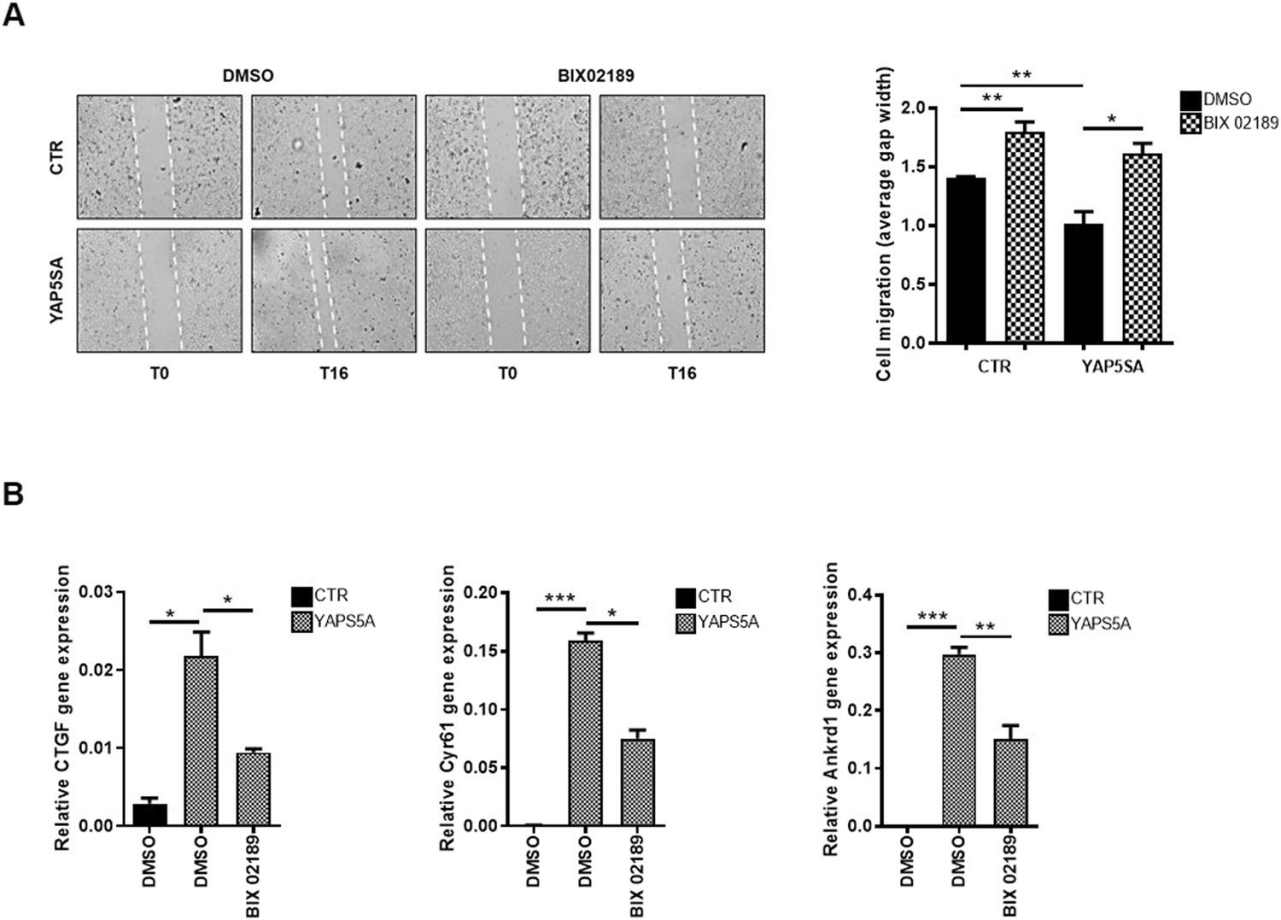
ERK5 inhibition interferes with YAP-dependent migration and gene expression of liver cancer cells in a LATS-independent manner. **A** Wound healing assay of YAP5SA-expressing HuH7 cells and control cells treated with 10 µM BIX02189 or DMSO for 16 h (T16). The images are representative of two independent experiments performed in duplicate. Cell migration was manually quantified as average distance between the edges of the gap (gap width). **B** RT-qPCR analysis of the indicated YAP target genes in mutant YAP5SA-overexpressing HuH7 cells (YAP5SA) and in control cells (CTR), treated with 10 µM BIX02189 or DMSO. Data are expressed as relative gene expression and shown as mean ± S.E.M. of three independent experiments. Statistically significant differences are reported (*p < 0.05; **p < 0.01; ***p < 0.001).

The key role played by YAP in cell migration, both in development and in cancer metastasis, has been well documented [1, 38, 48], but the molecular mechanisms involved in its regulation remain poorly described [49]. We therefore tested the role of ERK5 in the YAP-induced migration of HuH7 cells that display, together with an epithelial phenotype, a low rate of motility. To this end, HuH7 cells were transfected with a constitutively active mutant form of YAP (Supplementary Fig. S3A, B, right panels) and their migration has been assessed in a wound healing assay in the presence of the MEK5/ERK5 inhibitor BIX02189. As shown in Fig. 7A, the YAP-induced motility was significantly reduced by ERK5 inhibition, indicating the migration of tumor cells as a relevant functional readout of YAP regulation by ERK5. Interestingly, because the constitutively active mutant form of YAP utilized in the migration assay, named YAP5SA, carries five amino acid substitutions that make YAP non-phosphorylatable by LATS1/2 [50], these results indicate that ERK5 can regulate YAP activity in a Hippo/LATS-independent manner. This has been confirmed at molecular level since ERK5 inhibition can interfere with the YAP5SA-dependent gene expression (Fig. 7B).

Overall, these results, while not entirely excluding that ERK5 could also interfere with the canonical pathway of Hippo, point to ERK5 as a new element of the Hippo/LATS-independent regulation of YAP-driven transcription and cellular outcomes.

## Discussion

In the last few years, there has been a growing interest in the identification and characterization of new regulators of YAP activity, in order to unravel the increasingly evident complexity of extracellular stimuli and cellular outcomes mediated and driven, respectively, by this transcriptional cofactor. Mounting evidence indicates that an intertwined network of molecules and signaling can integrate the Hippo-pathway, considered the main YAP regulator, by converging on it at different levels or by directly controlling YAP activity in a Hippo- and LATS-independent way [51]. The knowledge of the key components of these regulatory networks and of their functional role has particular relevance in the perspective of identifying new therapeutic targets and setting up new protocols for the treatment of YAP-dependent pathologies.

In this study, we unveiled ERK5/MAPK as a new regulator of YAP transcriptional activity. In particular, by means of both ERK5 chemical inhibition and silencing experiments, we demonstrated that ERK5 activity is required for (i) the maintenance of YAP-dependent gene expression both in liver stem cells and in hepatocellular carcinoma cell lines, (ii) the YAP activation in cell adhesion dynamics and TGFβ-induced EMT, and (iii) the YAP-dependent cell migration.

Furthermore, we demonstrated that ERK5 signaling modulates the activity also of a YAP mutant non-phosphorylatable by LATS1/2, thus providing evidence of its, at least in part, independence from the core elements of the Hippo pathway.

Firstly, we would point out the reliability of the results obtained with the use of chemical inhibitors. Although both ERK5 inhibitors utilized in this work are not fully specific and some off-targets have been reported, the genetic silencing of ERK5 as well as its overexpression/constitutively activation should reassure about the relevance of this kinase in the processes described here. Moreover, regarding XMD8-92, we can reasonably rule out the involvement of its other target BRD4 (member of the BET family). This factor, in fact, acts on YAP-dependent transcription as a chromatin remodeling factors’ recruiter, and consequently it cannot be involved in the strong reduction of YAP activity that we observed in the chromatin-free luciferase assays presented in this work. Moreover, BRD4 is recruited on regulatory elements of YAP-target genes by a physical interaction with chromatin-bound YAP/TAZ [52]. After treatment with XMD8-92 we observed not only a reduction of YAP target gene expression, that could be referred also to BRD4 inhibition, but also a lack of YAP recruitment on DNA indicating at least a further, BRD4-independent, effect of the inhibitor. Furthermore, the modulation of YAP target genes has been obtained also with another chemical inhibitor, BIX02189, that does not interfere with BRD4 activity.

The here collected results are in line with and provide a mechanism for the correlation between ERK5 and YAP activity, previously described to exert a pivotal role in several cellular processes, primarily in cancers. The dysregulation of both ERK5 and YAP, indeed, has been related to increased metastatic risk and less favorable survival outcome [2, 22]. In particular, in hepatocellular carcinoma, YAP is present in an active form in more than 85% of tumor samples [53] and its inhibition restores hepatocyte differentiation and induces tumor regression in preclinical models [54]. On the other hand, ERK5/MAPK7 gene has been found amplified in primary HCC tumors [23] and the MEK5/ERK5 signaling pathway constitutively activated and associated with tumor growth [24]. Here, we showed that ERK5 is necessary and sufficient to induce YAP-dependent gene expression in HCC cell lines, thus identifying YAP as a new target of ERK5 in cancer. Furthermore, we showed that ERK5 activity is required for hepatoma cell migration induced by the constitutive activation of YAP.

Interestingly, both proteins have been demonstrated to be directly involved in the EMT [31, 55], a process crucial in carcinoma metastasization. The forced expression of YAP in cancer cells is sufficient to induce EMT, thus fostering tumor progression [56, 57]. Moreover, YAP acts as a primary mediator of TFGβ-induced EMT [33, 34] and is required for the SMAD2/3 nuclear translocation [35]. Furthermore, in liver stem cells, we showed that YAP is required for the maintenance of the mesenchymal state and that the expression of a constitutive active YAP in epithelial cells induces EMT-related gene expression through the direct and opposite transcriptional regulation of the EMT and MET master genes (i.e. Snail and HNF4α, respectively) [39]. We also demonstrated that MEK5/ERK5 signaling is required for Snail protein stabilization in hepatocytes undergoing EMT following TGFβ treatment [47]. Here, we have extended these results suggesting that the new direct ERK5-YAP axis may contribute to the EMT process and identified a mechanism of action.

Data obtained in this work showed, indeed, that ERK5 regulates YAP activity by allowing its physical interaction with the transcriptional partner, TEAD4, and, consequently, its recruitment on target gene promoters. No change in YAP protein level or subcellular localization was observed, as well as in its paralogous TAZ expression (data not shown). Notably, the overall data reported here support the finding of an ERK5-dependent regulation of YAP/TEAD complexes (i.e. regulation of the YAP/TEAD-dependent reporter gene activity, activation of YAP/TEAD target genes, regulation of YAP/TEAD interaction). However, although the YAP-regulated gene expression is mainly accomplished through the DNA binding mediated by TEAD family members, increasing evidence of additional transcriptional partners of YAP and of their role in triggering cell type- and context-specific cellular responses has been collected [58]. Therefore, the possible regulation by ERK5 of other YAP-containing transcriptional complexes cannot be excluded. As a matter of fact, the role played by ERK5 in complex YAP-dependent cell functions (i.e. motility), presumably involving the coordinate expression of several genes, enforces the hypothesis of a possible regulation by ERK5 of different transcriptional complexes including YAP. Further studies will be required to confirm this hypothesis.

Further investigations will be also needed to deeper analyze the molecular mechanism underlying the regulation of YAP activity by ERK5. This, in fact, could require the ERK5 kinase activity (resulting in the direct phosphorylation of YAP protein or of its transcriptional partners), but also its ability to act as transcriptional cofactor. ERK5, in fact, shows a functional transactivation domain [16] capable of driving gene expression through the interaction with transcription factors on DNA [59]. Therefore, the kinase could participate in the formation/activation/stabilization of a ternary transcriptional complex together with YAP and TEAD on target gene enhancers or promoters.

The knowledge of the molecular mechanisms involved in the functional regulation by ERK5 of YAP-containing transcriptional complexes, especially in cancer cells and in stem cells, could help to unveil new therapeutic targets for cancer treatment and new tools in regenerative medicine.

## Materials and methods

### Cell cultures and treatments

Resident liver stem cells (RLSCs) and HepE14 hepatocytes are immortalized, and non-tumorigenic cell lines derived from murine liver explants at 14th days of development [60–62]. RLSC and HepE14 were grown as previously described [39].

The human liver carcinoma cell lines HuH7 and HepG2 were grown at 37 °C, in a humidified atmosphere with 5% CO2 on plastic (Corning) in Dulbecco’s modified Eagle’s medium (DMEM; Gibco-Life Technologies), supplemented with 10% fetal bovine serum (FBS), 2 mM glutamine (Gibco-Life Technologies) and antibiotics (Gibco-Life Technologies).

For the experiments in 3D cell culture, HuH7 cells were grown in a low attachment substrate (0.6% agar) until the formation of suspended aggregates/spheroids (24 h).

Where indicated, cells were treated with 10 µM ERK5 inhibitor XMD8-92 (Selleckchem, Selleck Chemicals GmbH), 10 µM or 20 µM MEK5 inhibitor BIX02189 (Selleckchem, Selleck Chemicals GmbH), 10 µM of YAP-TEAD inhibitor Verteporfin or 4 ng/ml of TGFβ1 (PeproTech Inc., Rocky Hill, NJ, USA) for the indicated time. As previously reported, hepatocytes utilized in this study undergo EMT following TGFβ treatment [63–65].

The number of viable cells upon 16 h of treatment with MEK5/ERK5 inhibitors has been analyzed by CellTiter 96® AQueous One Solution Cell Proliferation Assay (Promega), following the manufacturer’s protocol.

### Cell transfections

ERK5-overexpressing cells were obtained by transient transfection with pCMV-ERK5 (carrying the human ERK5 cDNA, kindly provided by J.E. Dixon) and with pCMV-MEK5DD (carrying a phosphomimetic mutant sequence of human MEK5 cDNA, kindly provided by C.J. Marshall). Control cell lines were obtained by transfection with the empty vector. Cells were transfected with Lipofectamine™ LTX Reagent with PLUS™ Reagent (Thermo Fisher Scientific), according to the manufacturer’s protocol, and collected 48 h after transfection. YAP-overexpressing cells were obtained by transient transfection with pQCXIH-Myc-YAP or pQCXIH-Myc-YAP5SA (gift from Kunliang Guan, Addgene plasmids # 33091 and # 33093) [50], respectively, using Lipofectamine™ LTX Reagent with PLUS™ Reagent (Thermo Fisher Scientific) according to the manufacturer’s protocol. Cells were collected 48 h after transfection or utilized for treatments. Notably, YAP5SA protein, carrying mutations of LATS1/2-dependent phosphorylation sites (S61A, S109A, S127A, S164A, S381A), results constitutively active [50].

### Luciferase assays

To analyze endogenous ERK5 and YAP activity, cells were plated in 60 mm plates and co-transfected by Lipofectamine™ LTX with PLUS™ Reagent (Thermo Fisher Scientific) according to the manufacturer’s protocol with the following construct: MEF2-luc reporter [40] or 8XGTIIC-luc reporter (gift from Stefano Piccolo; Addgene plasmid # 34615) [41] (1 μg), Renilla expression vector (0.2 μg), pcDNA3 empty vector (4 μg). After 24 h, cells have been moved into 12-well plates and treated with MEK5 or ERK5 inhibitors or with their solvent DMSO, where indicated. All treatments were performed in triplicate.

To analyze ERK5- and YAP-dependent transcriptional activity, cells were plated in 12-wells plates and co-transfected by Lipofectamine™ LTX with PLUS™ Reagent (Thermo Fisher Scientific) with the following constructs: MEF2-luc reporter or 8XGTIIC-luc reporter (0.5 μg), Renilla expression vector (0.1 μg), pCMV-ERK5/pCMV-MEK5DD (1.5 μg/0.5 μg) or the empty vector (2 μg). All transfections were performed in triplicate.

Luciferase activity was measured by using the Dual-Luciferase Reporter Assay System kit (Promega Corporation, Madison, WI), according to the manufacturer’s instructions and normalized for Renilla luciferase activity.

### Immunofluorescence staining

For indirect immunofluorescence analysis, cells were fixed in 4% paraformaldehyde, permeabilized with 0.1% Triton-X100, and incubated with mouse monoclonal α-YAP antibody (SC-101199, Santa Cruz Biotechnology, inc.; 1:50) or rabbit polyclonal α-ERK5 antibody (#3372, Cell Signaling; 1:50). Alexa CY3-conjugated secondary antibodies (1:400; Molecular Probes, Eugene, OR, USA) were utilized. Nuclei were stained with 4′,6-diamidino-2-phenylindole (DAPI; Calbiochem Merck, Darmstadt, Germany). Images were acquired and processed as previously described [66]. The same enhanced color levels were applied for all channels.

### Western Blot analysis

Cells were lysed in RIPA buffer containing freshly added cocktail protease inhibitors (Sigma-Aldrich, St. Louis, MO). Equal amounts of proteins were loaded on 8% (for the analysis of ERK5 phosphorylation) or 12% acrylamide gels and then transferred to a nitrocellulose membrane (Bio-Rad). Blots were probed with the following primary antibodies: mouse monoclonal α-YAP (SC-101199, Santa Cruz Biotechnology, 1:1000), rabbit polyclonal α-ERK5 (#3372, Cell Signaling; 1:500) and mouse monoclonal α-GAPDH (MAB374, Millipore Corp., Bedford, MA, USA; 1:1000). Blots were then incubated with HRP-conjugated species-specific secondary antibodies (Bio-Rad,Hercules, CA, USA), followed by Enhanced Chemiluminescence reaction (ECL, Bio-Rad Laboratories Inc., Hercules, CA, USA).

### RNA isolation and quantitative RT-PCR

Total RNAs were extracted with ReliaPrep™ RNA Miniprep Systems (Promega) according to the manufacturer’s protocol and reverse-transcribed using Biorad iSCRIPT cDNA Synthesis Kit (Biorad). cDNA was amplified by qPCR using GoTaq qPCR Master Mix (Promega Corporation, Madison, WI) in BioRad-iQ-iCycler. Relative amounts, calculated with the 2(−ΔCt) method, were normalized with respect to the housekeeping gene RPL34 (60 S ribosomal protein L34). The sequence of murine and human primers utilized are listed in Table 1 and Table 2, respectively.

**Table 1:** List of mouse primers used for RT-qPCR experiments.

Gene	Forward primer (5′-3′)	Reverse primer (5′-3′)
*Ctgf*	ATCATGCTCGCCTCCGTCGC	TAGCAGGCCGGGTGCAGAGA
*Cyr61*	AGAGGCTTCCTGTCTTTGGC	CCAAGACGTGGTCTGAACGA
*Ddit4*	GCCGGAGGAAGACTCCTCATA	CATCAGGTTGGCACACAGGT
*Erk5*	TCTGACTCTGCAGCCTGCCCC	GGTGCACTGGGCCCATCTCTG
*Rpl34*	GGAGCCCCATCCAGACTC	CGCTGGATATGGCTTTCCTA

**Table 2:** List of human primers used for RT-qPCR experiments.

Gene	Forward primer (5′-3′)	Reverse primer (5′-3′)
*CTGF*	AGGAGTGGGTGTGTGACGA	CCAGGCAGTTGGCTCTAATC
*CYR61*	AAGAAACCCGGATTTGTGAG	GCTGCATTTCTTGCCCTTT
*ANKRD1*	AGTAGAGGAACTGGTCACTGG	TGGGCTAGAAGTGTCTTCAGAT
*ERK5*	CTGTCTACGTGGTCCTGGAC	GCCTTGTCCAAGTCCAAGTC
*YAP*	GTCCCGAACCCCTGGTAATAG	GGCCCTGCTGACATGTTTCTT
*RPL34*	GTCCCGAACCCCTGGTAATAG	GGCCCTGCTGACATGTTTCTT

### RNA interference by short hairpin RNA (shRNA) and small interfering RNA (siRNA)

pSUPER-shERK5 vector encoding shRNA specific for ERK5 was constructed according to Brummelkamp et al., 2002 [67]. The target sequence in both mouse and human ERK5 mRNA was 5′-TGAGAACTGTGAGCTCAAG-3′. Cells were transfected with pSUPER-shERK5 or the empty vector (from OligoEngine, Seattle, WA, USA) and utilized after 48 h for the experiments. Knockdown efficiency was confirmed by Western Blotting and RT-qPCR.

For siRNA-based ERK5 and YAP silencing, cells were transfected with equal amounts (100 pmol) of ON-TARGET plus SMARTpool mouse ERK5 siRNAs (L-040333-00-0005, GE Healthcare Dharmacon, Lafayette, CO, USA), ON-TARGET plus SMARTpool mouse YAP1 siRNA (22601; GE Healthcare Dharmacon, Lafayette, CO, USA), siRNA_YAP_hsa (5′-GACAUCUUCUGGUCAGAGAdTdT-3′ + 5′-UCUCUGACCAGAAGAUGUCdTdT-3′) using Lipofectamine RNAiMAX (Invitrogen) reagent in OptiMEM following the manufacturer’s protocol. RNA and proteins were harvested and analyzed after 48 h. siRNA against GFP (Gene Pharma) or Silencer™ Negative Control No. 1 siRNA (#AM4611, Ambion) were utilized as negative controls.

### Chromatin Immunoprecipitation (ChIP)

Chromatin immunoprecipitation analysis was performed as previously reported [65] using 5 μg of the following antibodies for the immunoprecipitation: rabbit polyclonal α-YAP (H-125X, Santa Cruz Biotechnology Inc.), or rabbit monoclonal α-YAP (D8H1X, 14074, Cell Signaling) or the negative control rabbit IgG (12370, Millipore Corp., Bedford, MA, USA). Equal amounts of immunoprecipitated DNA and relative controls were used for qPCR analysis, performed in triplicate. The primers utilized are the followings: *Ctgf* promoter, forward 5′-CAATCCGGTGTGAGTTGATG-3′ and reverse 5′-GGCGCTGGCTTTTATACG-3′; *Neurogenin 1*, forward 5′-CCTCCCGCGAGCATAAATTA-3′ and reverse 5′- GCGATCAGATCAGCTCCTGT-3′. The promoter of *Neurogenin1*, a gene not expressed in liver cells, was used as negative control. qPCR analysis of the immunoprecipitated samples (IP) and of the negative controls (IgG) were both normalized to total chromatin input and expressed as (IP/IgG)/Input.

### Co-Immunoprecipitation assay

HuH7 cells were transfected by Lipofectamine 3000 (Invitrogen) with pQCXIH-Myc-YAP plasmid and treated with BIX02189 or DMSO at 24 h post-transfection. Cells were harvested 16 h after treatments and lysed in Triton lysis buffer (150 mM NaCl, 50 mM Tris–HCl pH 7.5, 2 mM EDTA, 1% Triton-X100, 10% glycerol) supplemented with protease and phosphatase inhibitors. For immunoprecipitation, protein extracts (1 mg) were precleared with protein protein G-Sepharose (GE Healthcare, Little Chalfont, Buckinghamshire, UK) for 1 h and then incubated with 5 µg of mouse anti-TEAD antibody (ab58310, Abcam) or with normal mouse IgG (12371, Millipore Corp., Bedford, MA, USA), at 4 °C overnight. Then, protein G was added and incubated for 2 h. The beads were then washed three times in NetGel buffer (150 mM NaCl; 50 mM Tris–HCl pH 7.5; 1 mM EDTA; 0.1% NP-40; 0.25% gelatin) and twice with Triton lysis buffer. The immune complexes were eluted and denatured with Laemmli buffer 1X. Total and immunoprecipitated proteins were resolved on SDS-PAGE and transferred to the nitrocellulose membrane. For immunoblotting, the following primary antibodies were used: mouse polyclonal α-YAP (SC-101199, Santa Cruz Biotechnology, inc.; 1:1000), mouse polyclonal α-TEAD4 (ab58310, Abcam, 1:500), mouse monoclonal α-tubulin (B-7, sc-5286, Santa Cruz Biotechnology, 1:1000) Blots were then incubated with HRP-conjugated species-specific secondary antibodies (Bio-Rad,Hercules, CA, USA) or Goat α-mouse IgG light-chain specific antibody (HPR conjugate, #91196, Cell Signaling Technology), followed by Enhanced Chemiluminescence reaction (ECL, Bio-Rad Laboratories Inc., Hercules, CA, USA).

### Adhesion assay

For adhesion assays, HuH7 and HepG2 cells were seeded in non-coated plates for 24 h, then trypsinized, maintained in suspension for 10′, and collected or plated in triplicate on collagen-coated plates in the presence of DMSO or 10 µM BIX02189. After 3 and 4 h, respectively, cells were harvested and analyzed for gene expression.

### Wound healing assay

For the migration assay, YAP5SA-expressing and parental HuH7 cells were plated at high density on 35 mm dishes. 24 h later, the confluent layer of cells was scratched with a sterile tip to create an artificial wound. After rinsing with PBS to remove unattached cells, a low serum medium (0.5% FBS) was added in the presence of 10 µM BIX02189 or DMSO for 16 h. Cell migration was then analyzed by an optical microscope. Images were captured by Optech Digital Camera (Optech Technology) and the distance between the edges of the wound was manually quantified and expressed as average gap width.

### Statistical analysis

Statistical significance was determined by one-tailed paired Student’s *t* test or one-sample *t* test using GraphPad Prism Version 5 (GraphPad Software). A *p* ≤ 0.05 was considered statistically significant (*p < 0.05; **p < 0.01; ***p < 0.001).

## Supplementary information

Supplementary Information

Supplementary Figure S1

Supplementary Figure S2

Supplementary Figure S3

Supplementary Figure S4

Uncropped WB

aj-checklist

## Data Availability

All data generated and analyzed during the current study are available from the corresponding authors upon reasonable request.
