# Supplementary material for: Extracellular signal-Regulated Kinase 5 (ERK5) is required for the Yes-associated protein (YAP) co-transcriptional activity
Source: Cell Death Dis. 2023 Jan 17;14(1):32. doi: 10.1038/s41419-023-05569-7 (PMC9845357; doi:10.1038/s41419-023-05569-7)
Supplement: Supplementary file 1 — Supplementary Information [file 41419_2023_5569_MOESM1_ESM.docx]

**Supplementary Information**

**Extracellular signal-Regulated Kinase 5 (ERK5) is required for the Yes-associated protein (YAP) co-transcriptional activity**

Francesca Ippolito, Veronica Consalvi, Valeria Noce, Cecilia Battistelli, Carla Cicchini, Marco Tripodi, Laura Amicone and Alessandra Marchetti

**Supplementary Figure Legends**

**Supplementary Figure 1. (A)** Luciferase assay. The 8xGTIIC-luc reporter was transiently co-transfected in RLSCs, together with a Renilla expression vector, in the presence of siYAP or siCTR. Luciferase activities were normalized for Renilla luciferase activity and expressed as arbitrary units. Statistically significant differences are reported (**p<0.01). **(B)** RT-qPCR analysis of the indicated genes in RLSCs treated with 20 µM BIX02189 or DMSO. The values are calculated by the 2(−ΔCt) method, expressed as fold change in gene expression versus the control (DMSO, arbitrary value=1) and shown as means ± S.E.M. of at least three independent experiments. Statistically significant differences are reported (*p<0.05).

**Supplementary Figure 2. ERK5 is required for steady-state and adhesion-induced YAP activity in HepG2 hepatoma cell line. (A)** Luciferase assay. MEF2-luc or 8xGTIIC-luc reporters were transiently co-transfected in HepG2 cells. Twenty-four hours post-transfection, cells were treated with 10µM BIX02189 or its solvent DMSO for 16h. Luciferase activities were normalized for Renilla activity and expressed as arbitrary units. Statistically significant differences are reported (*p<0.05; **p<0.01). **(B)** RT-qPCR analysis of the indicated genes in HepG2 treated with BIX 02189 or DMSO. The values are calculated by the 2(−ΔCt) method, expressed as means ± S.E.M. of at least three independent experiments. Statistically significant differences are reported (*p<0.05). **(C)** RT-qPCR analysis of the indicated genes in HepG2. Cells were trypsinized, maintained in suspension for 10’ and collected (Susp) or attached for 4 hours on collagen-coated plates (Adh) in the presence of BIX02189 or DMSO. Statistically significant differences are reported (*p<0.05).

**Supplementary Figure 3. (A)** Western blot for the indicated proteins in YAP wild-type (left panel) or YAP5SA mutant (right panel) overexpressing HuH7, treated for 16h with DMSO or 10 µM BIX02189. GAPDH has been utilized as loading control. **(B)** Immunofluorescence analysis of HuH7 cells as in (A). Cells were stained with an anti-YAP antibody (red) and DAPI (nuclei, blue). Images are representative of three independent experiments. Scale bar: 50 µm.

**Supplementary Figure 4.** Dose-response analysis of ERK5 activity and cell proliferation in RLSCs (left) and HuH7 (right) cells treated with XMD8-92 and BIX02189, respectively. ERK5 activity has been assessed by luciferase assay with the MEF2-luc. Cell proliferation has been assessed by CellTiter 96® AQueous One Solution Cell Proliferation Assay. All analyses have been performed at 16 h of treatment.
