# Supplementary figures and images for: Extracellular signal-Regulated Kinase 5 (ERK5) is required for the Yes-associated protein (YAP) co-transcriptional activity
Source: Cell Death Dis. 2023 Jan 17;14(1):32. doi: 10.1038/s41419-023-05569-7 (PMC9845357; doi:10.1038/s41419-023-05569-7)

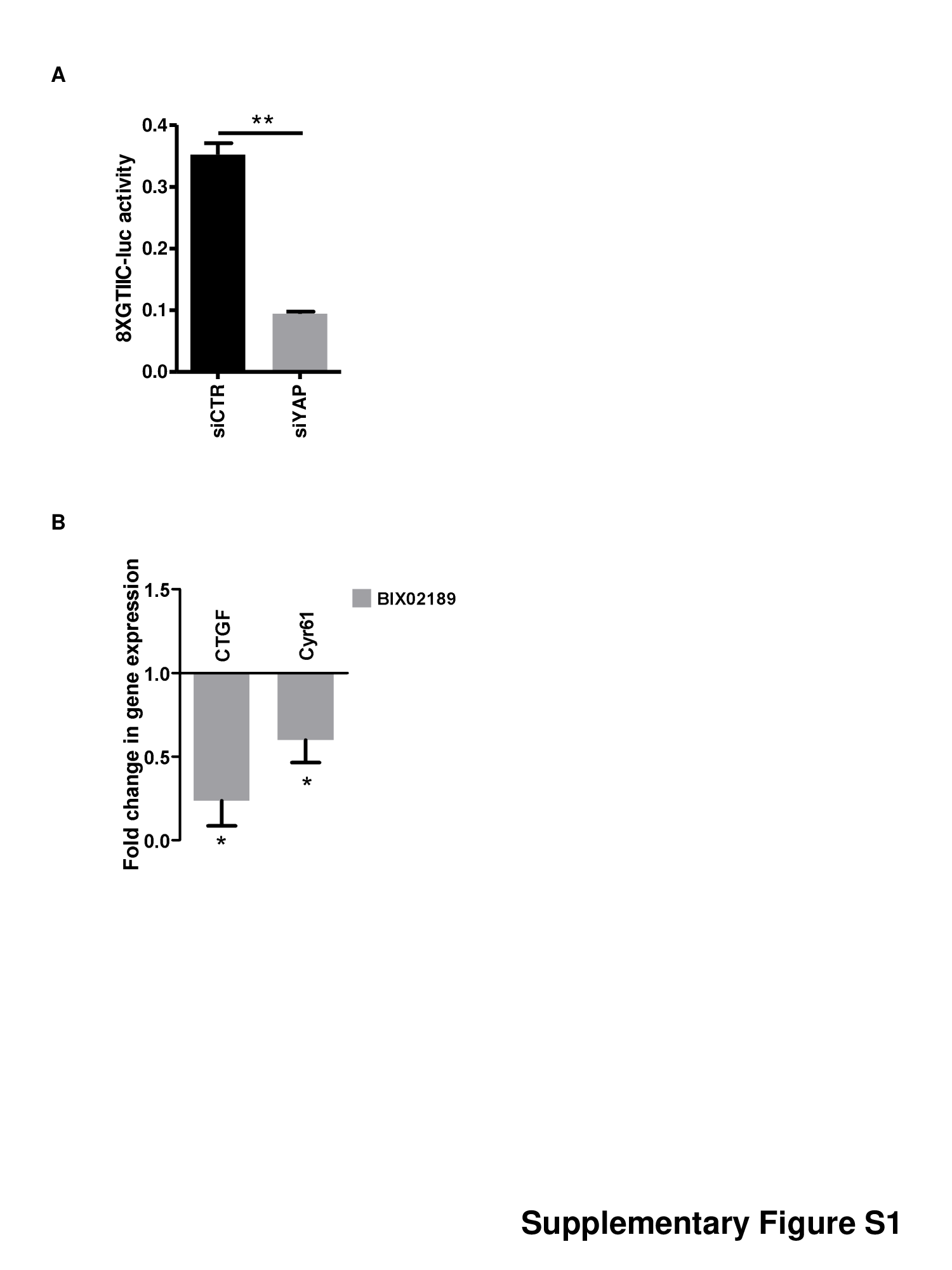

Supplement: Supplementary file 2 — Supplementary Figure S1 [file 41419_2023_5569_MOESM2_ESM.tif]

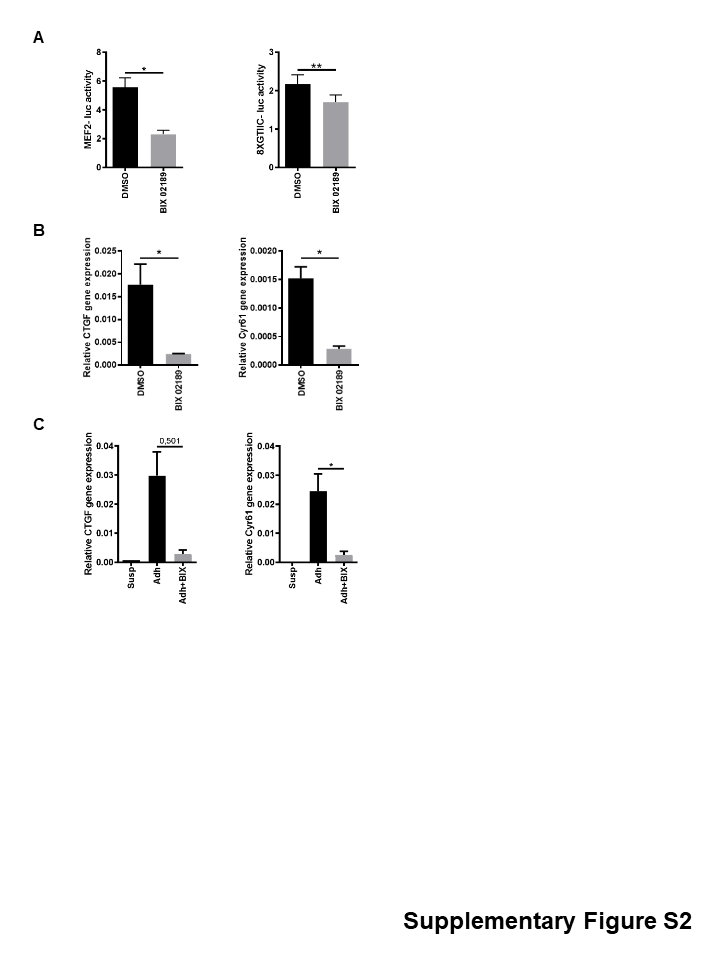

Supplement: Supplementary file 3 — Supplementary Figure S2 [file 41419_2023_5569_MOESM3_ESM.tif]

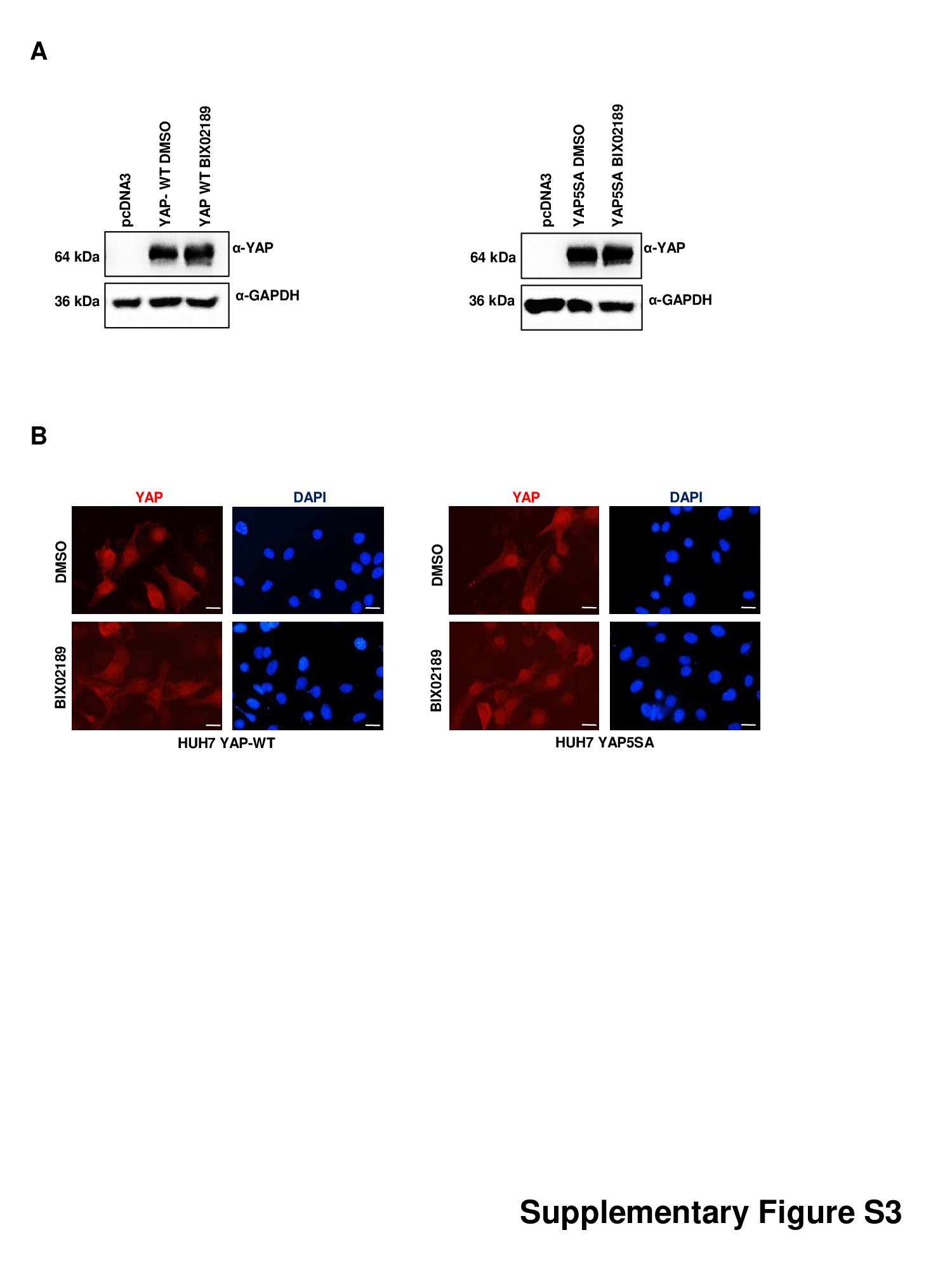

Supplement: Supplementary file 4 — Supplementary Figure S3 [file 41419_2023_5569_MOESM4_ESM.tif]

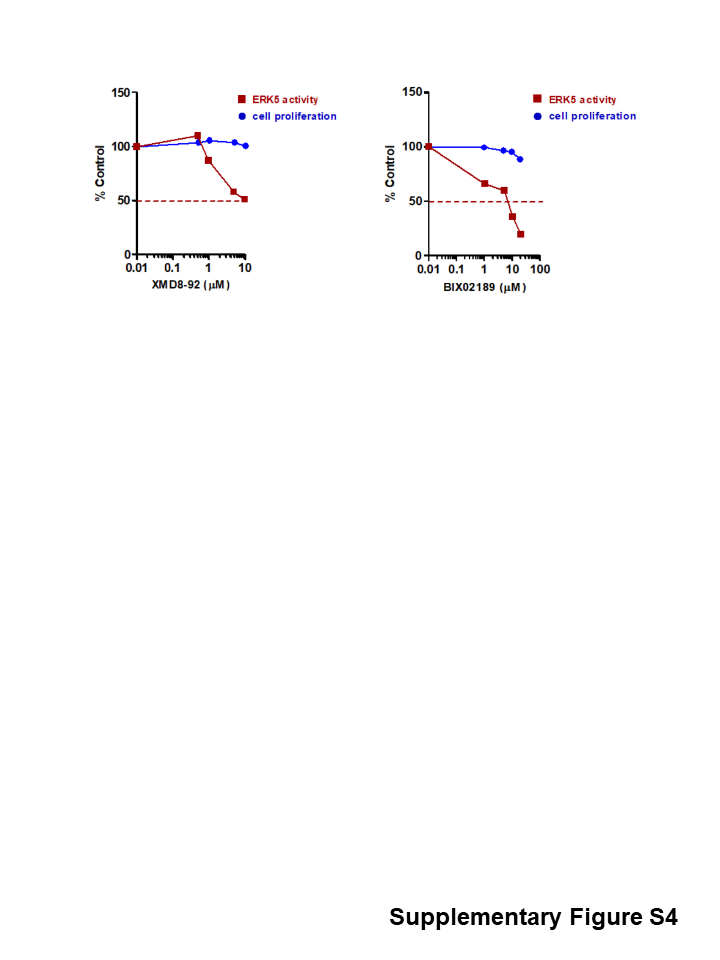

Supplement: Supplementary file 5 — Supplementary Figure S4 [file 41419_2023_5569_MOESM5_ESM.tif]

Figure 2E

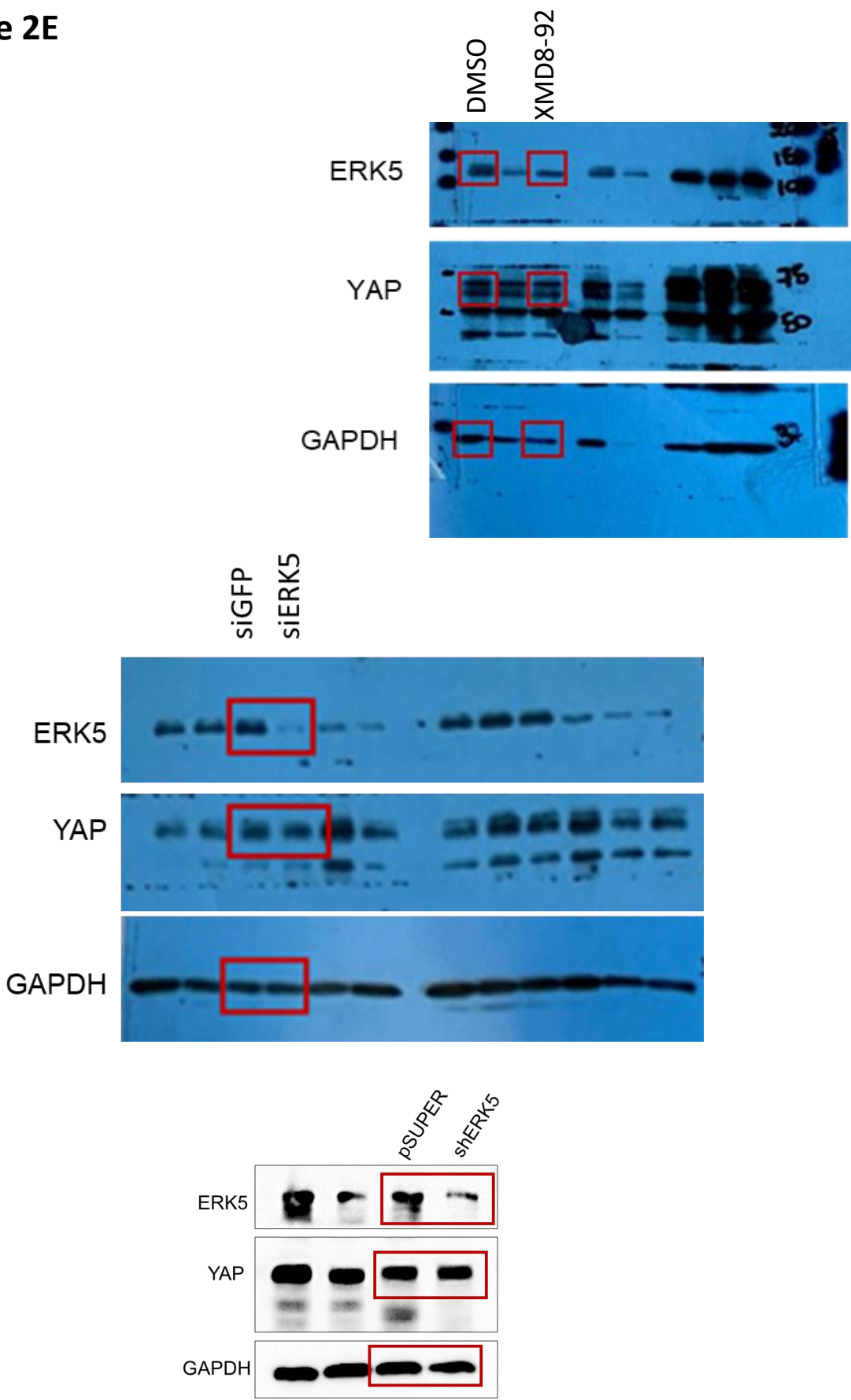

Figure 3B

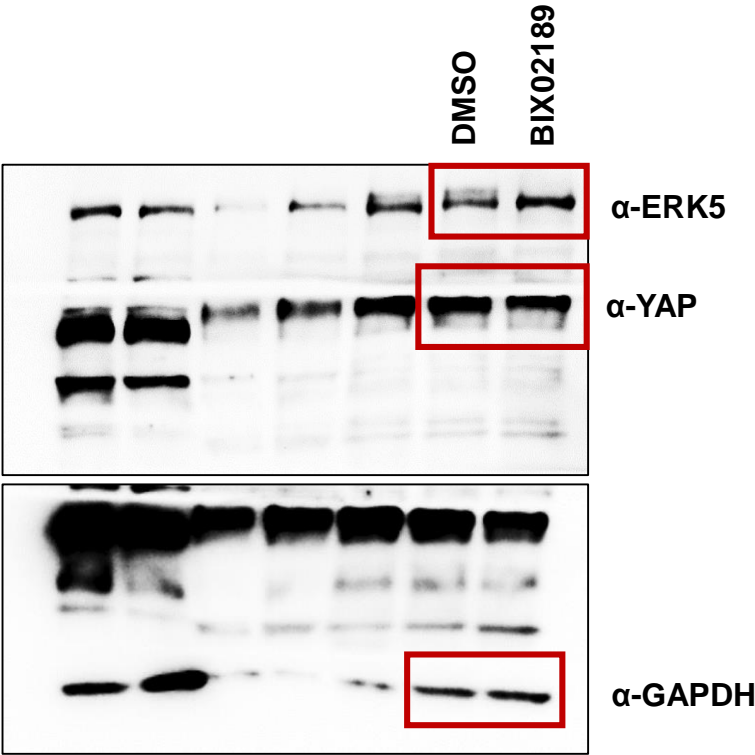

Figure 3D

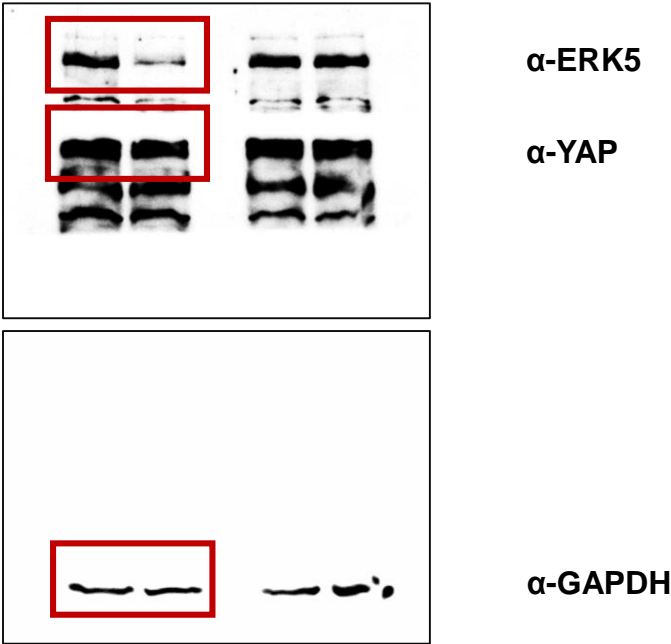

Figure 4B

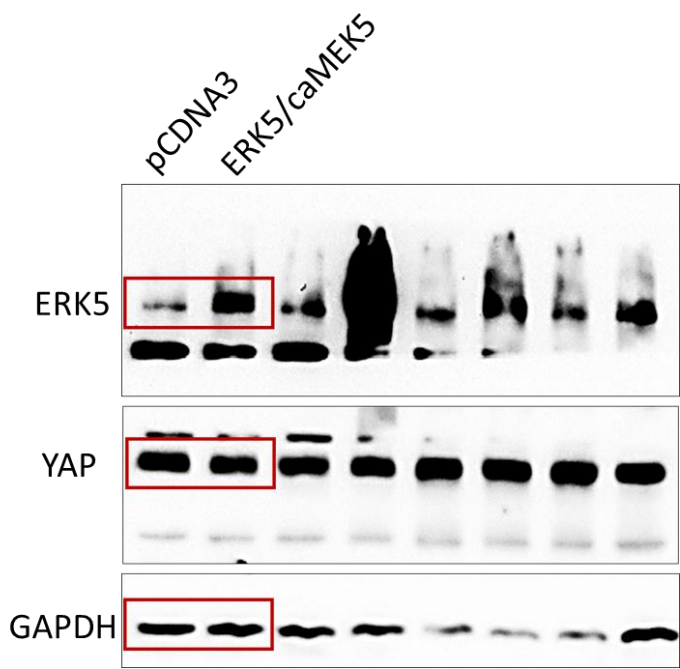

Figure 5D

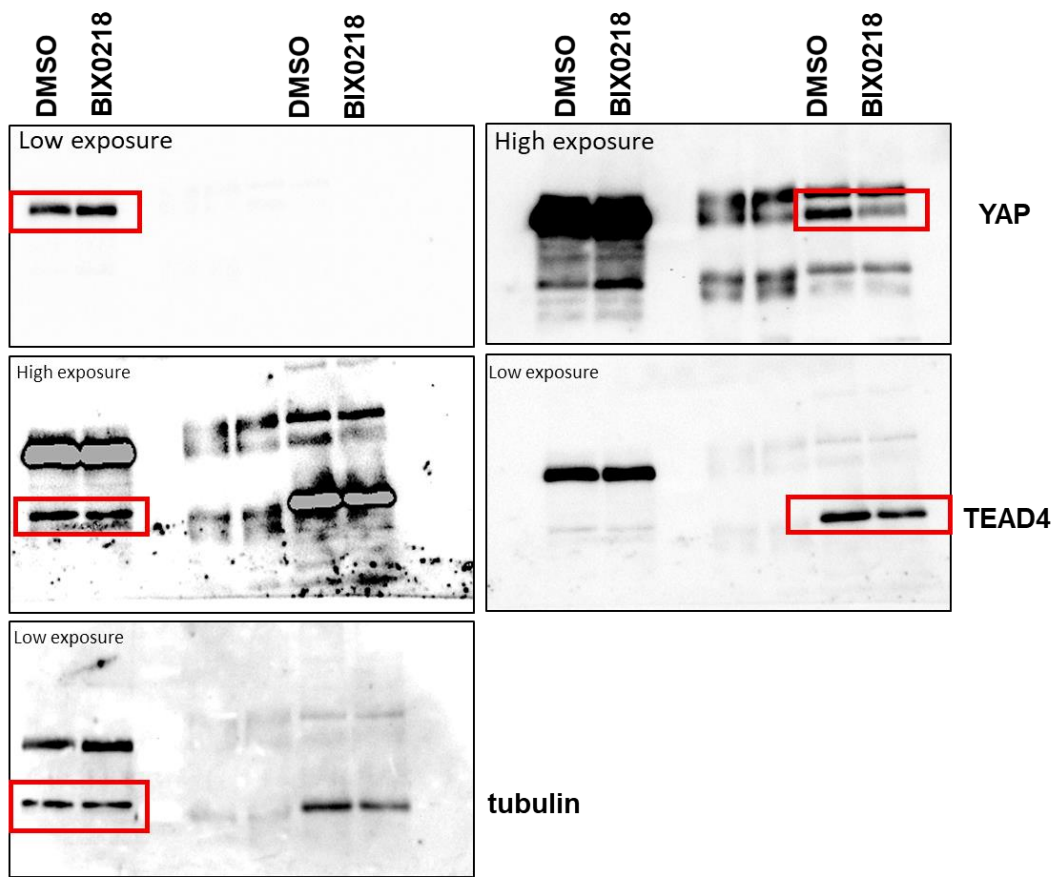

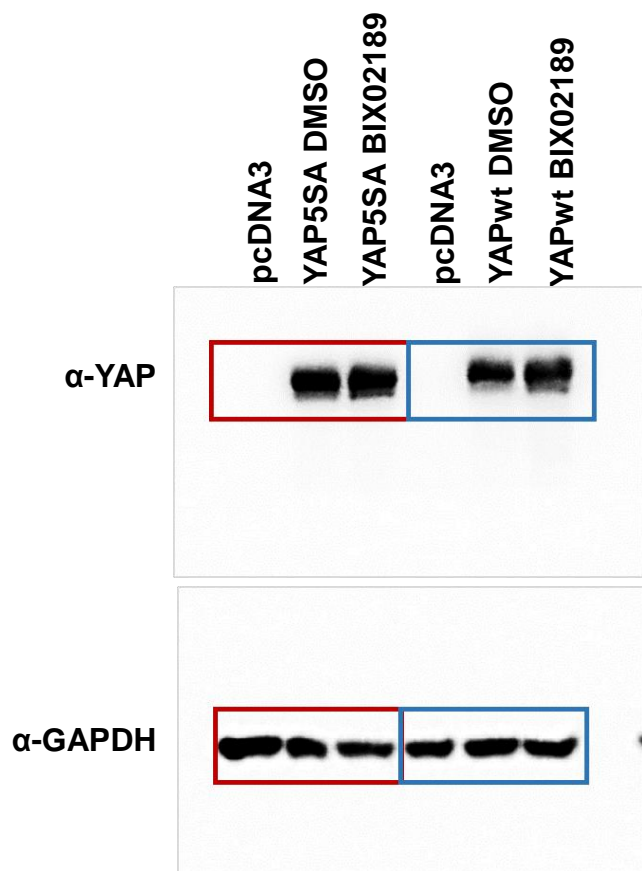

Supplement: Supplementary file 6 — Uncropped WB [file 41419_2023_5569_MOESM6_ESM.pdf]
